# Supplementary material for: Chromothripsis during telomere crisis is independent of NHEJ, and consistent with a replicative origin
Source: Genome Res. 2019 May;29(5):737–49. doi: 10.1101/gr.240705.118 (PMC6499312; doi:10.1101/gr.240705.118)
Supplement: Supplemental Material [file supp_gr.240705.118_Supplemental_file_1.zip › contigs/annotated_contigs/DB73/contig.2.DB73_length_271_mean_cov_3.87453874539.docx]

**DB73_length_271_mean_cov_3.87453874539**

```
TCTTTAAAATACTTTCTGAGGCTGCAAGGGTGCAAGG|GAAG|AGAAAGAAGGGGCCATGTCCCAGGAGCCCTTGCAACTGTCATCATT
 >chr5:141328828-141328869 + E=2e-13  >chr15:70429204-70429372 - E=6e-78
AGCTCTGTCCTTGTTTGTGTTTTTATTGCCTGTCTTCCCCCTGTAAAAGAAGCCATAGGACAGAAGAGGCCTTGGCCCCTTGCTTTCCT

GCTCTGCCTCCCAGGCCCACAACATTGCC|GGGCGAGCGGTGAGGATGCAAAGAGCATGTGTAGAGGAGAAGTGCTTGTGAATTGCGGT

TTGAATC
```
